# Cluster Analysis Statistical Spectroscopy for the Identification of Metabolites in ^1^H NMR Metabolomics

**DOI:** 10.3390/metabo12100992

**Published:** 2022-10-19

**Authors:** Silke S. Heinzmann, Melanie Waldenberger, Annette Peters, Philippe Schmitt-Kopplin

**Affiliations:** 1 Research Unit Analytical BioGeoChemistry, Helmholtz Munich, 85764 Neuherberg, Germany; 2 German Center for Diabetes Research (DZD), 85764 Neuherberg, Germany; 3 Research Unit Molecular Epidemiology, Institute of Epidemiology, Helmholtz Munich, 85764 Neuherberg, Germany; 4 German Center for Cardiovascular Disease Research (DZHK), Munich Heart Alliance, 80336 Munich, Germany; 5 Institute of Epidemiology, Helmholtz Munich, 85764 Neuherberg, Germany; 6 Institute for Medical Information Processing Biometry and Epidemiology (IBE), Ludwig-Maximilians-Universität München, 81377 Munich, Germany; 7 Chair of Analytical Food Chemistry, Technical University of Munich, 85354 Freising, Germany

**Keywords:** metabolomics, metabolite identification, urine, NMR spectroscopy

## Abstract

Metabolite identification in non-targeted NMR-based metabolomics remains a challenge. While many peaks of frequently occurring metabolites are assigned, there is a high number of unknowns in high-resolution NMR spectra, hampering biological conclusions for biomarker analysis. Here, we use a cluster analysis approach to guide peak assignment via statistical correlations, which gives important information on possible structural and/or biological correlations from the NMR spectrum. Unknown peaks that cluster in close proximity to known peaks form hypotheses for their metabolite identities, thus, facilitating metabolite annotation. Subsequently, metabolite identification based on a database search, 2D NMR analysis and standard spiking is performed, whereas without a hypothesis, a full structural elucidation approach would be required. The approach allows a higher identification yield in NMR spectra, especially once pathway-related subclusters are identified.

## 1. Introduction

Metabolomics deals with the comprehensive characterization of all small molecules, metabolites, in a biological system. It is the most downstream omics discipline and, therefore, the closest to the phenotype. While giving a good snapshot of end-points of metabolic processes, such as enzymatic reactions, it also provides insight into exogenous influences from the diet, drug intake and the gut microbiome. The concept is applied to many different biofluids, especially plasma, urine, stool extracts and saliva. Urine as the body’s waste product is not homeostatically controlled, and therefore, provides both temporal and chronic information of the metabolism from many different biological pathways and exogenous factors. In particular, it gives insight into dietary regimens, gut microbial composition and disease-related patterns. The chemical characterization of the whole metabolome is challenging from an analytical and chemical point of view. The selection of the analytical technique leads to some restrictions and pre-selection of detectable metabolites. The most widely applied techniques, to date, are mass spectrometry (MS) and nuclear magnetic resonance (NMR) spectroscopy, both having advantages and drawbacks. NMR spectroscopy is a particularly robust technique, offering direct quantification of elucidated metabolites and can be operated for both non-targeted and targeted metabolomics approaches. The robust and quantitative nature of the data makes the application of multivariate data analysis on the basis of correlation analysis particularly useful. Indeed, most statistical evaluations have focused on correlation analysis when interrogating NMR data [1].

With NMR spectroscopy being, historically, an indispensable tool for the structural elucidation of novel molecules, it provides a valuable tool for the identification of metabolites in non-targeted metabolomics analysis [2]. While many metabolites in urine and plasma are already known and identified [3,4], there are still many unknowns. Such unknown metabolites typically arise in studies where novel biomarkers for nutritional interventions, pharmacokinetics of medication and in relation to functional markers of the gut microbiome are sought. In this work, we report on a workflow for high-quality data curation and metabolite identification. In order to retrieve educated suggestions for compound identifications, we suggest applying cluster analysis statistical spectroscopy (CLASSY) [5], where correlation coefficients merge chemical shifts from the same metabolites into clusters, and hierarchical cluster analysis maps statistically related metabolite clusters together. Similar to statistical total correlation spectroscopy (STOCSY) analyses [6], such relationships are often biologically explainable, such as through the same pathways, the same dietary origin, etc. As an output, the peaks of the NMR spectrum are reordered from chemical shifts (i.e., electron susceptibility of each proton caused by surrounding electrons) to statistical relationships among all peaks.

We demonstrate that the application of both Spearman correlation and hierarchical clustering to the NMR dataset achieves the clustering of metabolites largely by their probable biological relationship. Which gives, in turn, valuable information for the further identification of unknown metabolites. Our report is useful in two ways, first, we give a list of commonly appearing microbial metabolites in urine NMR spectra that can serve as a database to the reader, and second, we give a detailed workflow description of the procedure enabling to reproduce our approach with other, large datasets for peak ID in new datasets.

## 2. Materials and Methods

### 2.1. Dataset Description

The KORA (Kooperative Gesundheitsforschung in der Region Augsburg) research platform has been collecting clinical and genetic data from the general adult population in the region of Augsburg, Germany for over 20 years [7]. F4 (2006–2008) and FF4 (2013–2014) cohorts are follow-up studies from the KORA S4 (*n* = 4261) survey carried out from 1999–2000. The KORA F4 cohort comprises 3080 subjects (aged 32–81 years), of whom 2161 participated in the follow-up study KORA FF4 (aged 39–88 years). For our sub-cohort analysis, we excluded participants with cancer, hepatitis and HIV infection, as well as samples with more than one freeze–thaw cycle. Further exclusion criteria were non-fasting on the interview day and the regular use of ibuprofen and/or paracetamol. A total of 998 samples have been selected randomly. In both studies, all participants have given written informed consent and the ethics committee of the Bavarian Chamber of Physicians, Munich, EC#06068) have approved the studies. Participants completed a lifestyle questionnaire, including details on health status and medication use in the past 7 days and underwent standardized examinations with urine samples taken [7]. All urine samples were stored at −80 °C until the analysis.

### 2.2. Sample Preparation 

Samples were defrosted on ice or kept in the fridge at 4 °C overnight, sorted in randomized order and homogenized by brief shaking. Aliquoting was performed by a Hamilton Microlab STAR^®^ (Hamilton Bonaduz AG, Bonaduz, Switzerland), where 150 µL urine samples were placed in each Eppendorf, followed by a 10 µL KF buffer (4.5 M KF in 100% D_2_O) and a 50 µL PO_4_ buffer (100% D_2_O, 0.1% TSP, 2 mM NaN_3_, pH 7.4). To create a study QC sample, a pool consisting of 10 µL of each urine sample in the study was collected. This QC was then thoroughly vortexed and aliquoted into 150 µL samples and mixed with the two above-mentioned buffers, and used for daily QCs. All samples were kept at −80 °C until analysis. Prior to NMR analysis, samples were defrosted on ice, vortexed, centrifuged (12,000 rpm for 10 min at 4 °C) and 180 µL was transferred to 3 mm outer diameter NMR tubes. For every day of analysis, one QC sample was additionally prepared.

### 2.3. NMR Analysis

Urine samples were analysed on a Bruker 800 MHz spectrometer operating at 800.35 MHz with a quadruple inverse cryogenic probe. A standard one-dimensional pulse sequence with water presaturation during the relaxation delay and the mixing time and the spoil gradient (noesygppr1d) was acquired with a recycle delay (d1) of 4 s, acquisition time (aq) of 3 s, mixing time (tm) set to 200 ms and a 90° pulse (p1) of 13 µs; 256 scans are collected into 64K data points with a spectral width of 16 ppm. NMR tubes were placed in the 4 °C cooled autosampler and quality checks were performed as follows: (i) weekly probe temperature control using 99.8% MeOD sample and tempcal function (300 K ± 0.003); (ii) daily shimming (TSP half width < 1.0 Hz) and water suppression quality using the QC sample. Phasing and baseline were also checked. Subsequently, all samples were submitted for analysis via a cooled autosampler. Once data acquisition was finished, all samples were briefly checked for phasing, baseline, water suppression and shimming and re-analysed if necessary. 2D NMR was performed on the QC sample and selected samples with a high concentration of peaks of interest. The 2D NMR analyses included J-resolved, TOCSY and HSQC, as previously described [8]. For TOCSY, 19,228 × 2048 datapoints were collected using 32 scans per increment and in the HSQC experiments 4788 × 1024 datapoints and 1024 scans per increment were collected.

### 2.4. NMR Data Processing

Acquired data were manually phased, baseline-corrected, calibrated to TSP (δ 0.00) using the TopSpin 3.6. (Bruker BioSpin) software. All spectra were imported to Matlab (Mathworks, Portola Valley, CA, USA), TSP peak and water peak residue were removed, resulting in the spectral area from 0.50–4.70 ppm and 5.00–9.50 ppm at a resolution of 0.00025 ppm (34,820 data points). Remaining positional noise of the peaks was overcome by a combination of local alignment of few distinct peaks and global alignment using the RSPA alignment method relative to the QC sample [9]. Spectra were then normalized using the probabilistic quotient normalization method [10]. The quality of the dataset was assessed by principal component analysis (PCA), where the focus was given to (i) drivers of the first principal components (i.e., exclude technical errors as a major driver of variation) and (ii) cluster of all QC samples relative to the whole dataset.

### 2.5. Clustering Approach

Cluster analysis statistical spectroscopy (CLASSY) according to Robinette et al. [5] was performed on the full dataset, consisting of a workflow with peak detection using the QC sample, followed by an automated identification approach of local and global clustering. For peak detection, a smoothed spectral derivative was calculated (Savitzky–Golay third-order polynomial filter with a window size 0.005 ppm) and peaks were detected at zero crossings [11]. Local clustering found structural correlation clusters based on Spearman correlation (cut-off 0.7) and global clustering applies to a hierarchical cluster analysis to arrange local clusters based on correlation clustering and average linkage. All settings were applied as previously described [5]. Peaks of the same molecule (‘structural affiliation’) clustered by local clustering (i.e., cut-off 0.7 for an indication as green boxes in the output graphic), and peaks or molecules from the same pathway clustered (‘biological affiliation’) close to each other in the global clustering (i.e., hierarchical cluster analysis and proximity of peaks along the axis).

### 2.6. Metabolite Identification

The CLASSY analysis was used to provide a guided suggestion for the affiliation of each peak to other peaks on the basis of correlation and cluster analysis. In some cases, we carried out STOCSY analysis to add statistical correlation information of the peak independent of peak picking. We also collected further information for every picked peak from the 2D experiments TOCSY and HSQC, i.e., their 1H-1H cross peaks and the 13C shift of the neighbouring C atom. When the picked peak is known, e.g., citrate with chemical shifts at δ 2.53 (d) and 2.67 (d) is present in every urine sample, we confirmed its identity using chemical shift, multiplicity, COSY or TOCSY analysis and HSQC analysis using information about shifts and multiplicities in databases such as HMDB [12]. When the identity of the peak is ad hoc not known, we need a hypothesis for the possible identity of the metabolite to avoid a full structural elucidation approach. The CLASSY approach gives an idea of the biological and structural nature of the compound giving rise to the chemical shift. Therefore, a hypothesis for its identity can be made and tested by 1D and 2D NMR, as previously described, and, where necessary, standard spiking experiments are carried out.

### 2.7. Quality Assessment of the Dataset 

**Figure 1:**
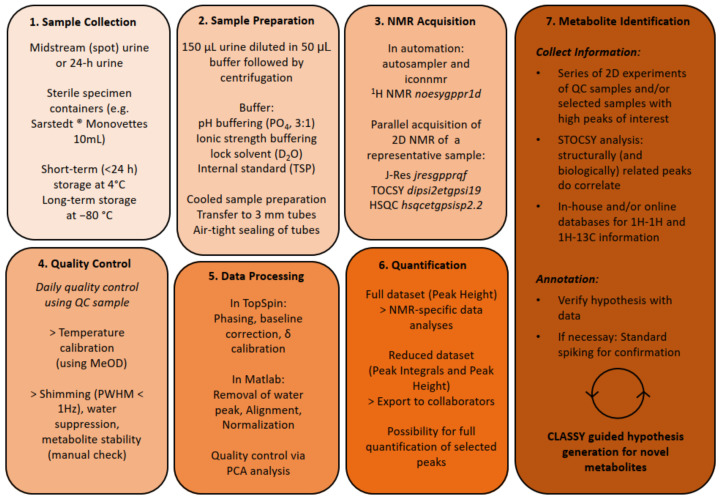
Overview of the standard operating procedures workflow.

A large and metabolically diverse dataset is necessary for assuring the presence of many different metabolites of different levels of concentration. We have chosen a subset of urine samples (*n* = 998) from the KORA epidemiological study. The stringent quality check parameters (see Section 2) retrieved high-quality spectra. This is a crucial part in data acquisition, especially when large batches of samples are measured or different batches and studies are combined post analysis. In order to gain an overview of the data quality and data structure, a principal component analysis (PCA) was performed. Both univariate and pareto scaled PCA (SI 1, Figure 1) retrieved a close clustering of the 21 QC samples, suggesting stability and absence of variation between measurement days. The main variation in the dataset (UV-scaling PC1 12.5, PC2 5.2%, pareto-scaling PC1 33.2%, PC2 9.2%) was attributed to biological variation, e.g., variation in the internal standard TSP (SI 1, i.e., arising indirectly from urine dilution) and glucose (SI 1, i.e., glucosuria). No apparent technical outliers (spectral quality (i.e., baseline variation, water suppression issues) and processing quality (i.e., alignment, normalization)) were apparent in the PCAs.

## 3. Results and Discussion

### 3.1. NMR Metabolomics Workflow and Metabolite Identification Approach

We established a metabolomics workflow that is suitable for large-scale studies. Urine samples are analysed following a strict standard operating procedure including sample collection, sample preparation, NMR acquisition, quality control and data processing considerations (Figure 1). It involves a cooled sample preparation (i.e., sample thawing and mixing of 150 µL sample, 50 µL phosphate buffer and 10 µL KF) [13] and analysis, and standardized NMR acquisition with daily quality control for instrument temperature and acquisition performance. Acquisition performance is monitored using a quality control (QC) sample concerning water suppression, shimming performance and sample stability.

**Figure 2:**
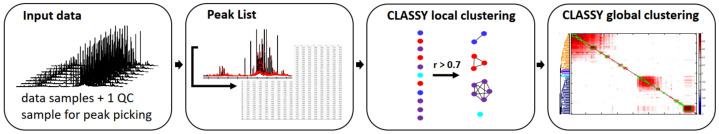
Overview of the CLASSY approach adopted from Robinette et al. Input data includes data samples and a QC sample. A peak-picking approach with the QC sample as reference generates the peak list. The CLASSY approach is comprised of a local clustering that finds peaks that cluster (Spearman correlation, see green boxes in the CLASSY clustering output figure also in Figure 3) and a global clustering based on HCA analysis for re-arrangement of the local clusters. The CLASSY output figure gives information on the peak clusters (green boxes), the exact correlation coefficient (colour-code red to blue) and a HCA dendrogram of all re-arranged peak clusters.

The processing of the samples involves pre-processing of the spectra, i.e., phasing, baseline correction and chemical shift calibration, water removal (4.7–5.0 ppm) RSPA alignment [9] and PQN normalization [10]. Subsequent quality assessment via PCA analysis is used for validating (i) a cluster of QC samples and (ii) biological variation is the main origin of main variation and no occurrence of technical outliers (spectral quality (i.e., baseline variation, water suppression issues) or lack of processing quality (i.e., alignment, normalization)), see also Supplementary Materials. Metabolites are relatively quantified by using the data point at peak maximum (i.e., signal intensity), and thereafter called “peaks”, or by applying a deconvolution algorithm which deconvolves and integrates peak areas under the curve [14]. For the main CLASSY analysis, only data points at peak maximum were used. Whereby peaks were selected by a peak-picking algorithm (see Section 2) based on the quality control (QC) sample, which is a mixture of all submitted samples, and therefore, theoretically contains all molecules of the dataset. The chosen peak-picking algorithm settings retrieved 846 peaks, which was an excellent trade-off between avoiding the selection of baseline, while finding most peaks above an S/N threshold of 5. In order to identify all known metabolites in the NMR spectrum, we manually listed chemical shift, multiplicity, 1H–1H cross peaks and 1H–13C cross peaks and information from STOCSY analysis of all peaks. For identification, we included information from the HMDB database [15], overview articles on the urine metabolome [3] information from the targeted platforms from Nightingale Health, Lifespin GmbH and Bruker IVDr urine and original articles that discuss methods and applications of NMR-based urine metabolomics [16,17,18,19]. Still, many peaks remained without identification. We faced three main issues: (i) database search by chemical shift suggested too many possibilities for manual inspection of all proposals; (ii) the confidence level for the identification of singlets is very low, having only one 1H and the corresponding 13C shift with often no or low statistical correlation from STOCSY analysis; (iii) even though it had multiple peaks belonging to one metabolite (i.e., as seen by TOCSY), we were not able to guess the structure nor identify the metabolite. Therefore, we propose to add another level of information, which is cluster analysis statistical spectroscopy (CLASSY) [5], introduced by Robinette et al. This approach comprises (i) peak picking, (ii) Spearman correlation-based local clustering and (iii) hierarchical clustering (HC) based global clustering (Figure 2).

**Figure 3:**
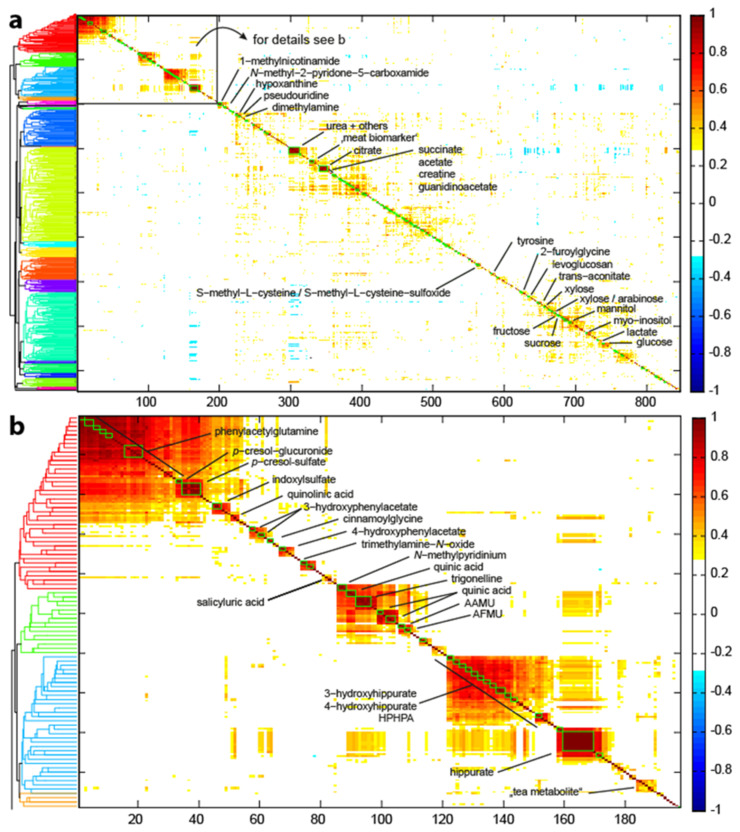
(**a**) Overview of all metabolite correlations from the 846 picked peaks. The x and y axis give the position of the peak in the metabolite correlation CLASSY cluster. Superimposed are the correlation coefficients, ranging from −1 to 1 (−1 (blue) to 1 (red), the area with small correlation coefficients, i.e., −0.3 to 0.3 is excluded (white)). The dendrogram (colour threshold 0.45) on the left highlights clusters. (**b**) The microbial metabolite cluster (from box in (**a**)) summarizes metabolites from amino acid breakdown (red dendrogram branch), from coffee consumption (green branch) and hippurate and hydroxy-hippurate metabolites (blue branch). The chemical shifts of the associated metabolites are listed in Supplemental Table S1.

The goal of this approach is to meaningfully re-arrange NMR peaks, which then give insights into their structural and biological relationships based on statistical metrics. The CLASSY approach works particularly well with large and diverse sample sets. Our dataset included 998 samples from the epidemiological study KORA. The local clustering arranged the 846 peaks into 635 local clusters, which were re-arranged in the global clustering by hierarchical cluster analysis employing correlation distances and average linkage (Figure 3), as also suggested and discussed by Robinette et al. Other distance and linkage criteria were compared; however, they retrieved inferior results (data not shown). Of the 635 clusters, 135 contained two or more peaks, while 500 clusters contained one peak. Owing to peak overlap and some remaining positional noise, the Spearman correlation coefficient of some peaks arising from the same molecule are below 0.7. However, such peaks mostly appear next to each other in the HCA dendrogram for global clustering. Similar effects were also observed when reducing the correlation threshold: peaks that were not defined as local clusters remained next to each other in the global clustering. Detailed inspection of the HCA cluster analysis revealed several observations: the main cluster (Figure 3b) contained many identified metabolites that were previously discussed to be of microbial origin, we therefore call this cluster “microbial cluster”. The other cluster contained mainly metabolites of endogenous origin, such as amino acids and TCA metabolites. Dietary metabolites were spread throughout the dendrogram. In the microbial cluster, several but not all metabolites were known. We hypothesized that the remaining unknown metabolites are metabolites of microbial origin, as discussed ahead.

### 3.2. Metabolite Identification Approach

The main separation in the HCA dendrogram contained known microbial metabolites (Figure 3b). The first part of this cluster (Figure 3, red dendrogram branch) assembles mainly metabolites from the microbial breakdown of the aromatic amino acids phenylalanine, tyrosine and tryptophan. These are the well-reported metabolites, phenylacetylglutamine, *p*-cresolsulphate and indoxylsulphate. Furthermore, we identified p-cresol-glucuronide (δ 5.09 (d)) as an alternative conjugate of p-cresol. The chemical shifts δ 6.80 (d) and δ 6.88 (d) derived from 3-hydroxy-phenylacetate and 4-hydroxy-phenylacetate, respectively. All other chemical shifts that belong to these two molecules are clustered directly next to each other (see Supplemental Table S1). Both metabolites are known to derive from both dietary intake and are associated with various gut bacteria. While 3-hydroxy-phenylacetate is linked to the precursor rutin [20], a flavonoid found in different plants, 4-hydroxy-phenylacetate is mainly associated to the intake of whole-grain consumption [21]. Both compounds are markers of gut Clostridium species [22,23]. Quinolinic acid (δ 7.45 (dd)), a reportedly mammalian breakdown product of tryptophan via kynurenine pathway also clustered here [24]; trimethylamine-N-oxide (δ 3.27 s), a microbial breakdown product of choline [25] and cinnamoylglycine (δ 6.73 d), did too. Interestingly, cinnamoylglycine is known to derive from plant cinnamates [26]; however, Bar et al. [27] reported good prediction of cinnamoylglycine levels in plasma by microbiome data and not diet data. Lastly, salicyluric acid (δ 7.80 dd, 7.02 m, 7.49 ddd), which can derive both from acetylsalicylic acid (Aspirin^®^) intake and consumption of vegetables [28], completed this cluster. The second subcluster (Figure 3, green dendrogram branch) was characterized by metabolites deriving from coffee consumption. These were N-methylpyridinium, quinic acid and trigonelline. Furthermore, four unknown peaks (δ 9.056 (s), 2.191 (s), 3.205 (s), 2.177 (s)) occurred in this subcluster.

This led us to speculate that they are related to coffee consumption. Two-dimensional NMR analysis showed no cross-correlation in TOCSY. HMDB database search within an error window of 0.01 ppm retrieved 244 hits. However, the top two hits (with one of the two shift matches) were xanthine derivate, which coincides with the lack of cross-correlations in TOCSY, as caused by heteroatoms in the purine ring. None of the expected caffeine derivates (i.e., caffeine, theobromine, paraxanthine, theophylline, 1,7-dimethyluric acid, 1,3,7-trimethyluric acid) matched the chemical shifts from chemical databases and standard spiking experiments. However, 5-acetylamino-6-amino-3-methyluracil (AAMU) matched the standard spiking into the urine, and the chemical shifts δ 2.177 and 3.205 were assigned to AAMU. 5-acetylamino-6-formylamino-3-methyluracil (AFMU) standard was not available for purchase. Based on spectra prediction of chemical shifts and biological relationship of AAMU to AFMU [29], we speculate that AFMU and several other xanthine derivatives (e.g., δ 3.38 (s), 7.85 (s), 3.00 (s), 8.02 (s), 7.93 (s), 3.32(s)) are also present in the urine samples. Two other metabolites, characterized by the chemical shifts 1.08 (d), and 2.42 (m) and 1.03 (d) and 2.55 (m), respectively were assumed to be short-chain aldehydes, which are also metabolites found in coffee. However, the standard spiking of isobutyrylaldehyde and isovaleraldehyde did not match the chemical shifts. The blue subcluster (Figure 3), that encompassed hippurate, its hydroxy-metabolites (3-hydroxyphenyl)-3-hydroxypropionic acid (HPHPA), 3-hydroxyhippuric acid (3-HHA) and 4-hydroxyhippuric acid (4-HHA)) and several unidentified metabolites. Based on the chemical shift ranging from δ 6.37 to 6.97 ppm, we assumed that the remaining unidentified metabolites are likely to be of polyphenolic origin and related to the consumption of tea, fruits and vegetables. Chemical shifts and splitting patterns suggest *m*-coumaric acid, *p*-coumaric acid, 3,4-hydroxyphenyl and pyrogallol. However, the available standard chemical compounds did not exactly match, which is likely due to differences in the conjugation (e.g., with acetate and sulphate). A yet unassigned metabolite is known to derive from the consumption of Earl Grey tea [30]. Taken together, we think that this subcluster contains metabolites of microbial polyphenol breakdown. We observed a strong inter-correlation of all protein-derived gut microbial metabolites, of all coffee metabolites and of all hippurate metabolites among themselves. Hippurate correlated also with many coffee metabolites, with cinnamoylglycine and 3-hydroxy-phenylacetate but not with protein-derived gut microbial metabolites. In summary, we identified 82% of the chemical shifts in the microbial cluster (161 of 197 peaks). For another 12% (36 peaks) we are confident to classify them into a compound class (i.e., xanthines and benzol derivates).

### 3.3. Validation and Performance

We evaluated the performance of our CLASSY approach by (i) modifying the NMR peak list and (ii) introducing additional independent datasets hence varying the input data (see Figure 2). First, we modified the peak list, i.e., the list of chemical shifts of the NMR signals of metabolites by using an alternative peak picking approach (i.e., based on local maxima [14]), where *n* = 1250 instead of *n* = 846 peaks were picked. We assessed the validity of the approach by comparing if the microbial cluster and if its subclusters were still grouped. Indeed, the clustering persisted. Furthermore, we did another modification of the peak list by drastically reducing the number of peaks, i.e., to *n* = 133 (only main peak of “identified metabolites”). We still obtained a clustering of most microbial metabolites (except for 3-hydroxy-phenylacetate and trimethylamine-*N*-oxide). We, therefore, can conclude that the CLASSY approach also works with alternative peak lists; however, it is beneficial to include a relatively large number of peaks without the previous exclusion of peaks.

Second, we extended the dataset to samples from other studies, e.g., measured years later with the same standard operating procedures protocol. These metabolomics studies included *n* = 57 urine samples from chronic kidney disease (CKD) study [31] and a flaxseed dietary study [32] on healthy individuals with *n* = 27 samples. Here again, the microbial cluster and its subclusters remained. On a similar note, we reduced the sample set step-wise by one hundred, down until *n* = 300 samples the clusters remained stable. However, the complete CLASSY cluster dendrogram required > 700 samples to be stable. We, therefore, advice to use large diverse datasets such as the KORA dataset as a basis for large metabolic diversity and embed future studies with smaller sample sizes.

## 4. Conclusions

In conclusion, we describe a SOP workflow for the analysis, processing and metabolite identification of urine samples for non-targeted metabolomics analysis using high-field NMR spectroscopy. We suggest CLASSY analysis as an additional instrument for the NMR metabolite identification toolbox. The CLASSY analysis approach utilizes correlation analysis and cluster analysis and rearranges NMR peaks based on their statistical similarity to each other. We show that the statistical similarity is largely caused by biological relationships, e.g., from shared pathways or biological and chemical classes. As an example, we give the microbial metabolites and their subclasses. These suggested affiliations allow an informed and narrower search in databases and a selection of chemical compound spiking experiments. We describe the workflow in detail to enable a widespread application for other users. However, the CLASSY analysis requires large and diverse datasets as input. Smaller (e.g., <300 samples) or biased datasets (e.g., nutritional interventions, strong disease impact, multiple sampling of few individuals) will introduce correlations based on the bias rather than a biological pathway or other classifications.

## Data Availability

The informed consent given by KORA study participants does not cover data posting in public databases. However, data are available upon request by means of a project agreement from KORA https://helmholtz-muenchen.managed-otrs.com/external, (accessed on 9 August 2022) and are subject to approval by the KORA Board.

## Supplementary Materials

The following supporting information can be downloaded at: https://www.mdpi.com/article/10.3390/metabo12100992/s1. Figure S1: quality assessment of the dataset; Figure S2: Salicyluric acid; Figure S3: Experimental details for sample preparation and acquisition of NMR spectra; Table S1: Table with metabolite annotations.
