# Supplementary material for: Cluster Analysis Statistical Spectroscopy for the Identification of Metabolites in 1H NMR Metabolomics
Source: Metabolites. 2022 Oct 19;12(10):992. doi: 10.3390/metabo12100992 (PMC9607017; doi:10.3390/metabo12100992)
Supplement: Supplementary file 1 [file metabolites-12-00992-s001.zip › metabolites-1882954-supplementary.pdf]

Supplemental Information 1: Quality assessment of the dataset

PCA analysis was carried out to investigate the main variations in the dataset (Figure 1). We used the dataset with chemical shifts ranging from -0.5 to 9.5 ppm and excluded the water peak between 4.7-5.00 ppm. The two first components (PC1 12.5 and PC2 5.2%) showed the internal standard TSP (see Figure 2, e.g. urine dilution) and urinary glucose (Figure 2, i.e. glucosuria) as main contributors of variation. Similarly, pareto-scaled PCA analysis revealed the same drivers in the principal component, the percentage of variation was higher (PC1 33.2%, PC2 9.2%). We did not observe evidence for technical outliers such as spectral quality (i.e. baseline variation, water suppression issues) and processing quality (i.e. alignment, normalization)).

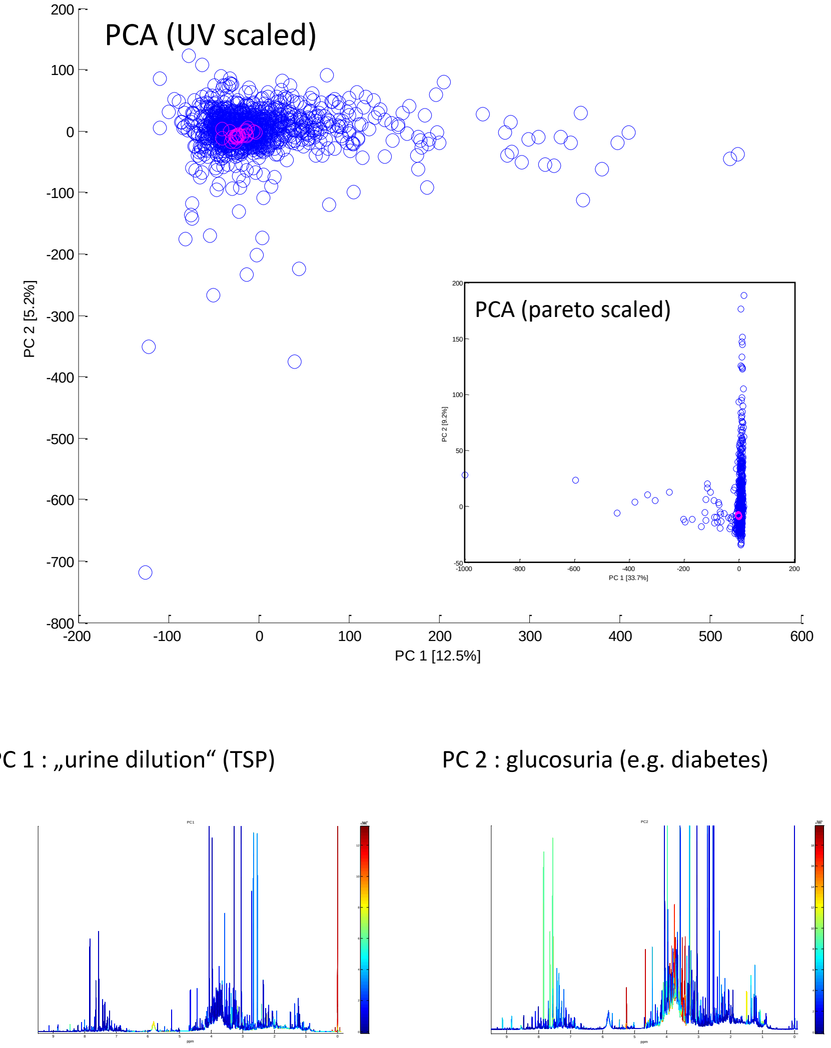

SI Figure S1: PCA analysis showing main contribution in the PCA plots (both univariate and pareto scaling) from amounts of internal standard TSP (relative to metabolites, showing the urine dilution) and glucose (highlighting glucosuria mainly from diabetes patients).

Supplemental Information 2: Salicyluric acid

In order to confirm the identity of salicyluric acid (SUA) we conducted a spiking experiment of the chemical standards and salicylic acid (SA) into pure urine. Figure 1 shows the pure standards and spikings in different concentrations. We conducted the spiking as a series spiking, i.e. analysis of pure urine, then spiking of the first standard (5µL SUA) with immediate analysis, followed by spiking of 5 µL SA and analysis. Lastly, we respiked 10 µL of SUA to reconfirm a further rise of the resonances seen after 5 µL of SUA. Indeed, chemical shifts of SUA and SA are similar but distinguishable and match the chemical shifts seen in our dataset.

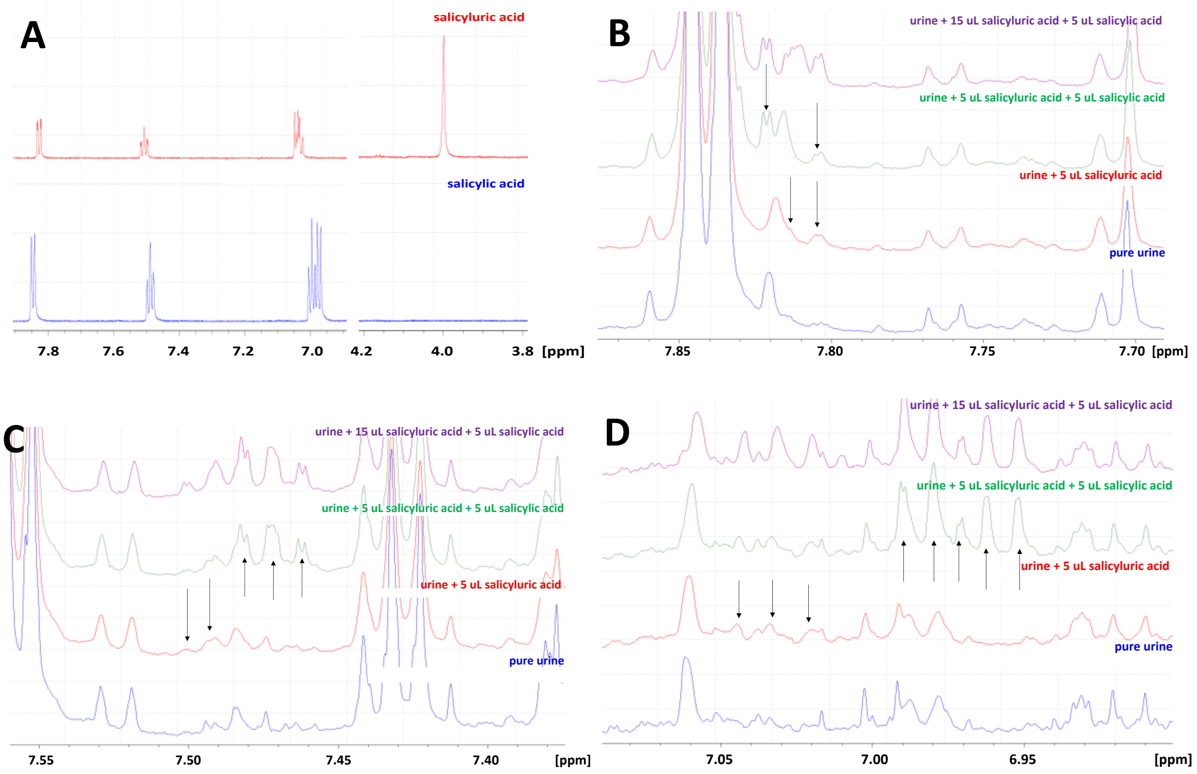

Figure S2: Spiking experiment of salicyluric acid and salicylic acid to human urine (QC sample). (**A**) pure standard samples of salicyluric acid and salicylic acid, (**B-D**) 1H NMR spectra of pure urine and urine with addition of 5 uL salicyluric acid (0.5 mg/mL), 5 uL salicylic acid (1.2 mg/mL) and 15 uL salicyluric acid (0.5 mg/mL) + 5uL salicylic acid (1.2 mg/mL) with highlight of the appearing resonances by arrows.

OPLS-DA analysis of per n-fold plot identified SUA excreters vs all other individuals highlighted all resonances from SUA and additional resonances from glucuronidated salicylic acid metabolites and paracetamol.

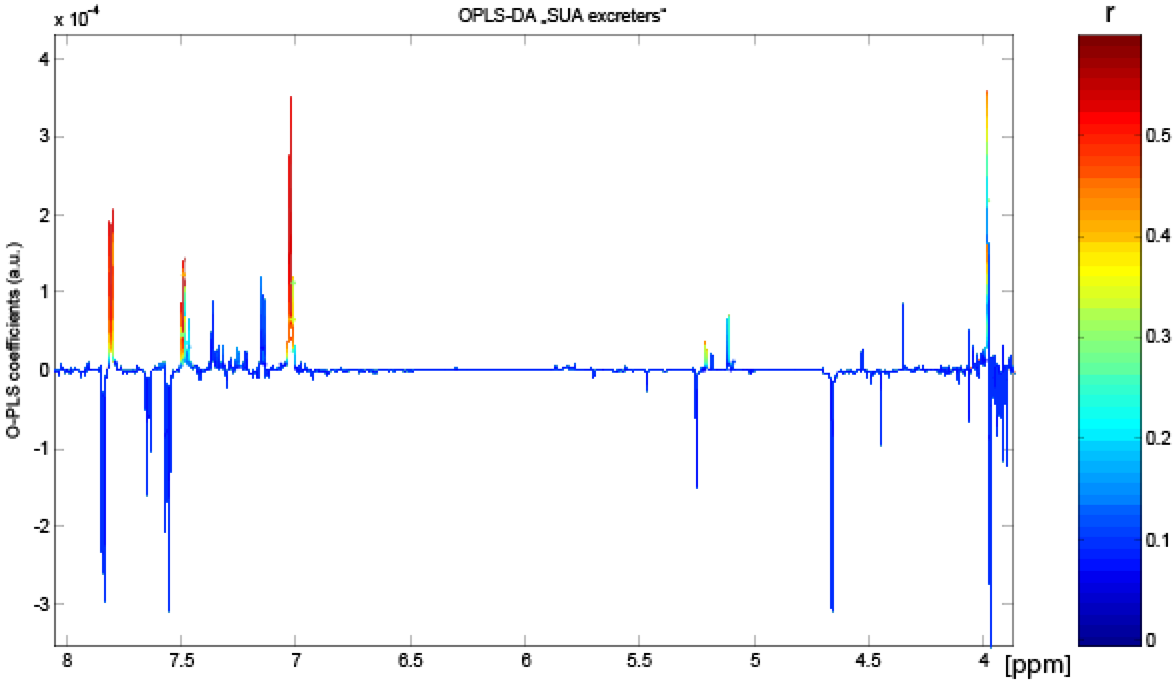

Figure S3: Loadings plot of OPLS-DA analysis comparing SUA excreters with all other individuals. THE SUA resonances (δ 7.806 dd, 7.49 ddd, 7.022 m, 3.98s ppm). Additional resonances appear at δ 5.11 (d) and 5.21 (d).

Supplemental Information 3: Experimental details for Sample preparation and acquisition of NMR spectra

Automated sample preparation protocol for urine samples

- Overnight thawing in the fridge at 4 °C
- Vortex sample
- Transfer of 150 µL to Eppendorf using Hamilton Microlab STAR® liquid handler
- Simultaneous production of QC:
    - 5 µL of every sample to pool
    - Vortex and split into “samples” of 150 µL each (n = 1000 samples -> 5 mL -> 33 QCs)
- Addition of phosphate buffer (50 µL) and KF (10 µL):
    - PO_4 buffer: 100% D_2O, 0.1% TSP, 2 mM NaN_3, pH 7.4
    - KF buffer: 4.5 M KF in 100% D_2O
- Gentle mixing by 3x inversion
- Store in fridge till analysis (max 24 hours) or in freezer at -80 °C
- Vortex and centrifugation (12,000 rpm for 10 min at 4°C)
- Transfer to 3 mm NMR tubes
- Air-tight sealing
- Analysis by NMR spectroscopy

NMR spectroscopy

Pulse program: noesygppr1d

Size of fid: 64K

Dummy scans: 16

Scans: 256

Sweep width: 16 ppm

Acquisition time: 3s

Mixing time: 200 ms

90° pulse (p1): 13 µsec

Relaxation (d1): 4 s

Receiver gain: 45.2

Dwell time: 39 µsec

Line broadening: 0.3

Temperature: 300K

SI_Table_S1

#	ppm shift	metabolite annotation	HMDB ID
1	7.4400	phenylacetylglutamine	HMDB0006344
2	7.3548	phenylacetylglutamine	HMDB0006344
3	7.3790	phenylacetylglutamine	HMDB0006344
4	2.2760	phenylacetylglutamine	HMDB0006344
5	2.2658	phenylacetylglutamine	HMDB0006344
6	7.4308	phenylacetylglutamine	HMDB0006344
7	7.3633	phenylacetylglutamine	HMDB0006344
8	2.1208	phenylacetylglutamine	HMDB0006344
9	2.1033	phenylacetylglutamine	HMDB0006344
10	1.9328	phenylacetylglutamine	HMDB0006344
11	1.9243	phenylacetylglutamine	HMDB0006344
12	4.1695	phenylacetylglutamine	HMDB0006344
13	4.1745	phenylacetylglutamine	HMDB0006344
14	2.2848	phenylacetylglutamine	HMDB0006344
15	2.1135	phenylacetylglutamine	HMDB0006344
16	2.1090	phenylacetylglutamine	HMDB0006344
17	2.0933	phenylacetylglutamine	HMDB0006344
18	1.9518	phenylacetylglutamine	HMDB0006344
19	1.9413	phenylacetylglutamine	HMDB0006344
20	1.9150	phenylacetylglutamine	HMDB0006344
21	1.9048	phenylacetylglutamine	HMDB0006344
22	2.1265	phenylacetylglutamine	HMDB0006344
23	7.4213	phenylacetylglutamine	HMDB0006344
24	3.6815	phenylacetylglutamine	HMDB0006344
25	3.6580	phenylacetylglutamine	HMDB0006344
26	4.1833	phenylacetylglutamine	HMDB0006344
27	4.1900	phenylacetylglutamine	HMDB0006344
28	8.0023	phenylacetylglutamine	HMDB0006344
29	2.1365	phenylacetylglutamine	HMDB0006344
30	2.0993	phenylacetylglutamine	HMDB0006344
31	2.1308	phenylacetylglutamine	HMDB0006344
32	6.7863	phenylacetylglutamine	HMDB0006344
33	5.0910	p-cresol-glucuronide	HMDB0011686
34	5.0815	p-cresol-glucuronide	HMDB0011686
35	7.2915	p-cresol-sulfate	HMDB0011635
36	7.2815	p-cresol-sulfate	HMDB0011635
37	7.2223	p-cresol-sulfate	HMDB0011635
38	7.2188	p-cresol-sulfate	HMDB0011635

39	7.2083	p-cresol-sulfate	HMDB0011635
40	2.3478	p-cresol-sulfate	HMDB0011635
41	2.3008	p-cresol-glucuronide	HMDB0011686
42	3.6395	phenylacetylglutamine	HMDB0006344
43	7.9925	phenylacetylglutamine	HMDB0006344
44	4.1635	phenylacetylglutamine	HMDB0006344
45	7.7100	indoxylsulfate	HMDB0000682
46	7.5230	indoxylsulfate	HMDB0000682
47	7.5125	indoxylsulfate	HMDB0000682
48	7.7003	indoxylsulfate	HMDB0000682
49	7.3718	indoxylsulfate	HMDB0000682
50	7.2760	indoxylsulfate	HMDB0000682
51	7.4650	quinolinic acid	HMDB0000232
52	7.4553	quinolinic acid	HMDB0000232
53	7.4613	quinolinic acid	HMDB0000232
54	7.2330		
55	7.2285		
56	2.3420	p-cresol-sulfate	HMDB0011635
57	7.2575	3-hydroxyphenylacetate	HMDB0000440
58	7.2480	3-hydroxyphenylacetate	HMDB0000440
59	7.2543	3-hydroxyphenylacetate	HMDB0000440
60	6.8098	3-hydroxyphenylacetate	HMDB0000440
61	6.8020	3-hydroxyphenylacetate	HMDB0000440
62	7.2685	3-hydroxyphenylacetate	HMDB0000440
63	6.7430	cinnamoylglycine	HMDB0011621
64	6.7230	cinnamoylglycine	HMDB0011621
65	7.9080		
66	7.0420		
67	6.8810	4-hydroxyphenylacetate	HMDB0060390
68	6.8703	4-hydroxyphenylacetate	HMDB0060390
69	7.1748	4-hydroxyphenylacetate	HMDB0060390
70	7.1640	4-hydroxyphenylacetate	HMDB0060390
71	3.4525	4-hydroxyphenylacetate	HMDB0060390
72	3.3515		
73	3.2768	trimethylamine-N-oxide	HMDB0000925
74	7.4915		
75	7.4808		
76	7.4715		
77	7.3333		
78	7.3428		
79	7.8120	salicyluric acid	HMDB0000840

80	7.8000	salicyluric acid	HMDB0000840
81	7.4995	salicyluric acid	HMDB0000840
82	7.0313	salicyluric acid	HMDB0000840
83	7.0223	salicyluric acid	HMDB0000840
84	7.0158	tea metabolite	
85	7.0125	tea metabolite	
86	8.7970	N-methylpyridinium	HMDB0240545
87	8.7905	N-methylpyridinium	HMDB0240545
88	4.4060	N-methylpyridinium	HMDB0240545
89	1.8930	quinic acid	HMDB0003072
90	1.8795	quinic acid	HMDB0003072
91	1.8628	quinic acid	HMDB0003072
92	9.1280	trigonelline	HMDB00875
93	8.8505	trigonelline	HMDB00875
94	8.8413	trigonelline	HMDB00875
95	8.0950	trigonelline	HMDB00875
96	4.4465	trigonelline	HMDB00875
97	8.0858	trigonelline	HMDB00875
98	8.1038	trigonelline	HMDB00875
99	1.9798	quinic acid	HMDB0003072
100	1.9750	quinic acid	HMDB0003072
101	1.9835	quinic acid	HMDB0003072
102	1.9650	quinic acid	HMDB0003072
103	1.9600	quinic acid	HMDB0003072
104	1.9565	quinic acid	HMDB0003072
105	1.9713	quinic acid	HMDB0003072
106	9.0563	5-acetylamino-6-formylamino-3-methyluracil	HMDB0011105
107	2.1910	5-acetylamino-6-formylamino-3-methyluracil	HMDB0011105
108	3.2050	5-acetylamino-6-amino-3-methyluracil	HMDB0004400
109	2.1773	5-acetylamino-6-amino-3-methyluracil	HMDB0004400
110	1.8708	quinic acid	HMDB0003072
111	7.9363		
112	3.3238		
113	1.0825	unknown, coffee cluster	
114	1.0740	unknown, coffee cluster	
115	1.0323	unknown, coffee cluster	
116	0.9803		
117	8.2293		
118	3.3878		
119	7.8543	unknown, coffee cluster	
120	3.0028		

121	8.0268		
122	7.1305	3-hydroxyhippurate	HMDB06116
123	7.1275	3-hydroxyhippurate	HMDB06116
124	6.9898	4-hydroxyhippurate	HMDB13678
125	6.9800	4-hydroxyhippurate	HMDB13678
126	6.8643	(3-hydroxyphenyl)-3-hydroxypropionic acid	HMDB02643
127	6.8540	(3-hydroxyphenyl)-3-hydroxypropionic acid	HMDB02643
128	6.9300	(3-hydroxyphenyl)-3-hydroxypropionic acid	HMDB02643
129	5.0103	(3-hydroxyphenyl)-3-hydroxypropionic acid	HMDB02643
130	7.1215	3-hydroxyhippurate	HMDB06116
131	7.1185	3-hydroxyhippurate	HMDB06116
132	7.4110	3-hydroxyhippurate	HMDB06116
133	7.3035	(3-hydroxyphenyl)-3-hydroxypropionic acid	HMDB06116
134	3.9488	3-/4-hydroxyhippurate	HMDB06116/HMDB02643
135	2.6998	(3-hydroxyphenyl)-3-hydroxypropionic acid	HMDB02643
136	7.3135	3-hydroxyhippurate	HMDB06116
137	6.9270	(3-hydroxyphenyl)-3-hydroxypropionic acid	HMDB02643
138	2.6273	(3-hydroxyphenyl)-3-hydroxypropionic acid	HMDB02643
139	2.6198	(3-hydroxyphenyl)-3-hydroxypropionic acid	HMDB02643
140	2.6015	(3-hydroxyphenyl)-3-hydroxypropionic acid	HMDB02643
141	5.0278	(3-hydroxyphenyl)-3-hydroxypropionic acid	HMDB02643
142	5.0205	(3-hydroxyphenyl)-3-hydroxypropionic acid	HMDB02643
143	7.5918	unknown, hydroxyhippurate-cluster	
144	7.5815	unknown, hydroxyhippurate-cluster	
145	7.3235	3-hydroxyhippurate	HMDB06116
146	7.3100	3-hydroxyhippurate	HMDB06116
147	7.7465	4-hydroxyhippurate	HMDB13678
148	7.7350	4-hydroxyhippurate	HMDB13678
149	2.6895	(3-hydroxyphenyl)-3-hydroxypropionic acid	HMDB02643
150	2.6088	(3-hydroxyphenyl)-3-hydroxypropionic acid	HMDB02643
151	6.9943	4-hydroxyhippurate	HMDB13678
152	6.9835	4-hydroxyhippurate	HMDB13678
153	7.7570	4-hydroxyhippurate	HMDB13678
154	7.7680	4-hydroxyhippurate	HMDB13678
155	3.9443	3-/4-hydroxyhippurate	HMDB06116/HMDB02643
156	3.9530	3-/4-hydroxyhippurate	HMDB06116/HMDB02643
157	7.6043	unknown, hydroxyhippurate-cluster	
158	7.6328	hippurate	HMDB00714
159	7.5455	hippurate	HMDB00714
160	7.8448	hippurate	HMDB00714
161	7.8358	hippurate	HMDB00714

162	7.6558	hippurate	HMDB00714
163	7.6463	hippurate	HMDB00714
164	7.6370	hippurate	HMDB00714
165	7.5698	hippurate	HMDB00714
166	7.5595	hippurate	HMDB00714
167	7.5500	hippurate	HMDB00714
168	3.9753	hippurate	HMDB00714
169	3.9683	hippurate	HMDB00714
170	7.6605	hippurate	HMDB00714
171	8.5560	hippurate	HMDB00714
172	8.5455	hippurate	HMDB00714
173	7.8688	hippurate	HMDB00714
174	7.9403	hippurate	HMDB00714
175	7.9305	hippurate	HMDB00714
176	6.9675		
177	6.9625	sumiki's acid	HMDB02432
178	6.9575		
179	6.9470		
180	6.9365		
181	6.6065		
182	6.8975		
183	6.8875		
184	7.4008	tea metabolite	
185	4.5078	tea metabolite	
186	6.9730	tea metabolite	
187	7.0658	tea metabolite	
188	7.3963	tea metabolite	
189	7.3880	tea metabolite	
190	7.0725	tea metabolite	
191	7.1133	3-hydroxyhippurate	HMDB06116
192	7.1043	3-hydroxyhippurate	HMDB06116
193	6.6953		
194	6.6900		
195	6.6698		
196	6.3725		
197	6.3925		
